# Angiomotin like-1 is a novel component of the N-cadherin complex affecting endothelial/pericyte interaction in normal and tumor angiogenesis

**DOI:** 10.1038/srep30622

**Published:** 2016-07-28

**Authors:** Yujuan Zheng, Yuanyuan Zhang, Giuseppina Barutello, Kungchun Chiu, Maddalena Arigoni, Costanza Giampietro, Federica Cavallo, Lars Holmgren

**Affiliations:** 1 Department of Oncology and Pathology, Cancer Centrum Karolinska, Karolinska Institutet, SE-17176 Stockholm, Sweden; 2 Department of Molecular Biotechnology and Health Sciences, Molecular Biotechnology Center, University of Turin, 10126 Turin, Italy; 3 Department of Biosciences, Milan University, Via Celoria 26, Milan 20133, Italy and IFOM, the FIRC Institute of Molecular Oncology, Via Adamello 16, Milan 20139, Italy

## Abstract

Transmission of mechanical force via cell junctions is an important component that molds cells into shapes consistent with proper organ function. Of particular interest are the cadherin transmembrane proteins, which play an essential role in connecting cell junctions to the intra-cellular cytoskeleton. Understanding how these biomechanical complexes orchestrate intrinsic and extrinsic forces is important for our understanding of the underlying mechanisms driving morphogenesis. We have previously identified the Amot protein family, which are scaffold proteins that integrate polarity, junctional, and cytoskeletal cues to modulate cellular shape in endothelial as well as epithelial cells. In this report, we show that AmotL1 is a novel partner of the N-cadherin protein complex. We studied the role of AmotL1 in normal retinal as well as tumor angiogenesis using inducible endothelial-specific knock-out mice. We show that AmotL1 is essential for normal establishment of vascular networks in the post-natal mouse retina as well as in a transgenic breast cancer model. The observed phenotypes were consistent with a non-autonomous pericyte defect. We show that AmotL1 forms a complex with N-cadherin present on both endothelial cells and pericytes. We propose that AmotL1 is an essential effector of the N-cadherin mediated endothelial/pericyte junctional complex.

The formation of blood vessels demands an intrinsic synchronization of cellular events such as migration, formation of cellular contacts and establishment of cellular asymmetry. This is exemplified in vascular development by the highly coordinated formation of the axial vessels by the proliferation and migration of mesodermal progenitor cells to the body midline^1^. The circulatory network is further expanded through sprouting of endothelial cells that migrate and anastomose with neighboring sprouts. This process invokes the formation and the dynamics of cell-cell junctions, apical-basal polarity as well as the control of the cytoskeleton to modulate cell shape. Maturation of vessels also involves the reciprocal interaction between endothelial cells and pericytes which envelope the vascular tube.

An intriguing question in biology is how such clearly complicated signals are integrated to control blood vessel formation. Here, studies of the Angiomotin (Amot) scaffold protein family may provide some mechanistic insight. This protein family consists of three members Amot, AmotL1 and AmotL2 that localize to junctions and regulate cell growth and motility (Zheng *et al*. 2009)^2^. All three members share a N-terminal domain with PPXY protein interaction motifs, a coiled-coil domain and a C-terminal protein-binding motif. Several groups have shown that the Amot proteins associate to the Crb3 and Par3 apical polarity proteins via the PDZ binding motif^3^,^4^. The coiled-coil domain binds the tumor suppressor Merlin as well as the RICH1 GAP protein^4^,^5^. The N-terminal WW-binding motif binds the HIPPO effectors YAP1, MST2 and LATS2 and also mediates binding to the junctional protein MAGI1b and actin^6^,^7^. The identification of the Amot binding partners suggests a role of these scaffolds to integrate essential morphogenic cues to control cell shape and proliferation of multicellular tissues.

We have previously shown by gene inactivation in zebrafish and mouse models, that the three members of the Amot protein family exert distinct roles during blood vessel development^8^,^9^. AmotL2 associates with VE-cadherin in order to connect the adhesion junction complex to the actin filaments thus establishing a mechanical connection between endothelial cells^9^. Using traction force microscopy, we also demonstrated that AmotL2 is essential to generate force in endothelial junctions. This may explain the lack of aortic expansion when genetically interfering with AmotL2 expression during zebrafish or mouse development. The pivotal role of Amot in physiological angiogenesis was demonstrated by the fact that its inactivation results in inhibition of migration of inter-segmental vessels in zebrafish and also affects blood vessel development in causing death in utero^8^. Finally, recent data indicate that Amot, similarly to AmotL2, is also part of a junctional complex as it associates to CDH11 and may be involved in promoting migration in prostate cancer^10^. Less is known however regarding the role of AmotL1 in the formation and maintenance of cell-cell contacts. Morpholino knock-down experiments suggest a perturbed adhesion of stalk cells of inter-segmental vessels and some overlap in function with Amot (at the level of tight junctions)^8^,^11^. In this report, we have used an endothelial-specific knock-out approach to analyze the role of AmotL1 in normal and pathological angiogenesis in mice. We identify AmotL1 as a novel component of the N-cadherin adhesion protein complexes and provide evidence that AmotL1 is essential for vascular remodeling during angiogenesis.

## Results

In order to investigate the role of AmotL1 in the vascular endothelium and other tissues, we used a genetic deletion approach to silence gene expression in mouse. To inactivate AmotL1 in a cell-type specific fashion, we generated mice with loxP sites flanking exons 5 and 6 (Supplementary Figure 1a). The *amotL1*^flox/flox^ mice were mated with Cdh5 (PAC)-CreERT2 transgenic mice (hereafter abbreviated as a*motL1*^ec+/ec+^)^12^. This model allows the efficient tamoxifen-inducible conditional recombinase expression in the endothelial cell lineage (recombined mice are abbreviated *amotL1*^ec−/ec−^). Tamoxifen-induced recombination was verified by genomic PCR analysis (Supplementary Figure 1b). The efficiency of tamoxifen injections in inducing recombination events was also analyzed in tumors from MMTV-Cre/*amotl*^*f*lox/flox^/MMTV-PyMT mice by western blot (Supplementary Figure 1c).

**Figure 1:**
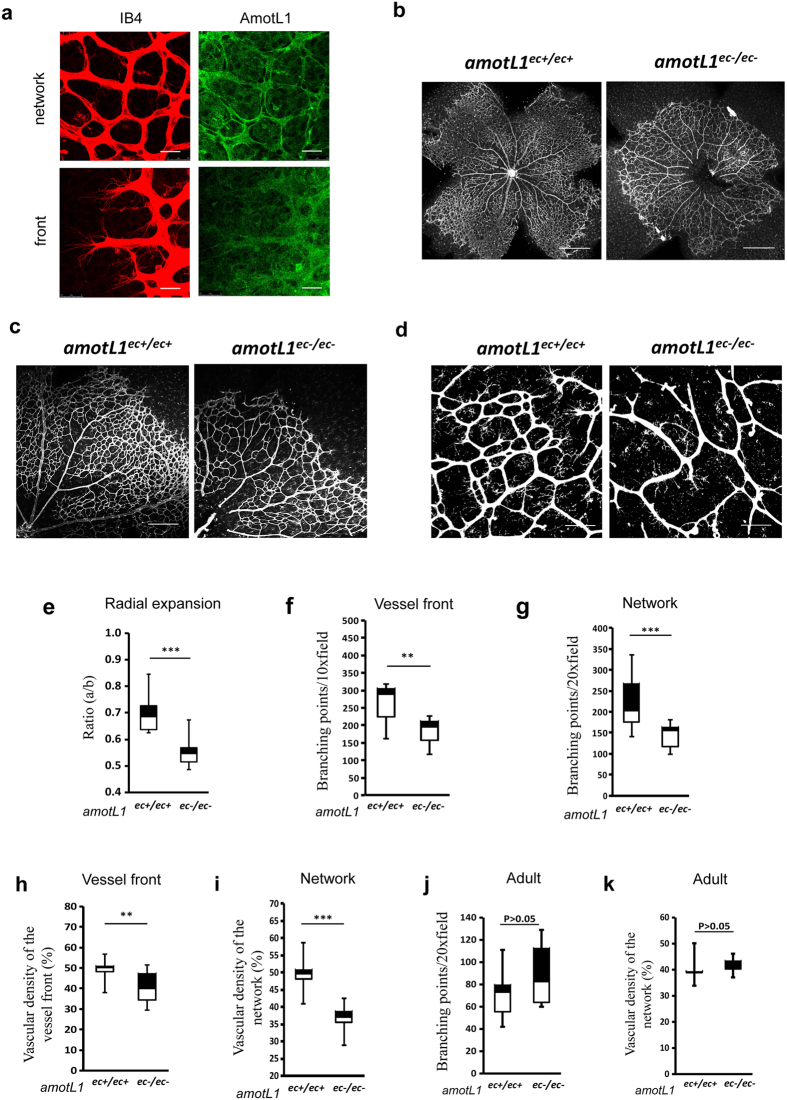
Defects in vessel formation in post-natal retinas of *amotL1*^ec−/ec−^ mice. (**a**) Whole-mount immunofluorescence (IF) staining of wild-type mouse retina at postnatal day 6 (P6). Blood vessels were visualized with isolectin B4 (IB4, in red) and AmotL1 using rabbit anti-AmotL1 polyclonal antibodies (in green). The upper panels display the central blood vessels network and the lower panels are derived from the front of the expanding vasculature. (**b–d**) Whole-mount IF staining of *amotL1*^ec+/ec+^ and *amotL1*^ec−/ec−^ mouse retinas at post-natal day 6 (P6). Blood vessels were visualized with IB4 lectin staining. (**e**) Radial vessel expansion was quantified by the ratio of vessel length divided by the length of the retina as measured from the optic nerve. (**f,g**) Bar diagrams show quantification of branch points at the vessel front or in the vessel network. (**h,i**) Bar diagrams show quantification of vascular densities at the vessel front or in the vessel network. (**j,k)** Quantification of branching points and vascular density in retinas from adult animals after genetic deletions was induced in the adult stage. ^**^P < 0.005; ^***^P < 0.001. Size bars are (a and d) 50 μm. (b) 1 mm. (c) 100 μm.

We analyzed effects of inactivation of AmotL1 protein expression during post-natal vascularization of the mouse retina. The retinas of newborn mice are avascular but become vascularized in a very reproducible manner over the first 10 days after birth^12^. In order to detect AmotL1 protein expression we have previously developed immunoaffinity-purified antibodies that did not cross-react to any of the other family members^11^. Western blot analysis showed expression of both AmotL1 isoforms (p90 and p100) in developing as well as in adult retina (Supplementary Figure 1d). We also used whole mount immunostaining of mouse retinas of post-natal day 6 to analyze AmotL1 expression. AmotL1 showed junctional localization in established vessels of the developing vascular network. No staining above background was detectable in endothelial cells of the front of the expanding vascular network (Fig. 1a).

We then analyzed effects of inactivation of AmotL1 protein expression during post-natal vascularization of the mouse retina. In *amotL1* inactivation experiments, pups were injected with tamoxifen on P1-3 and retinas were harvested on P6. The developing vasculature was visualized by whole mount staining using isolectin B4^12^. Overall, the density of the vessel network of the *amotL1* deficient retinas was reduced and covered a smaller area of the retina (Fig. 1b,e). The vascular density and the number of branching points, evaluated using the Angiotools program^13^, were significantly lower in the periphery and center of the *amotL1*^ec−/ec−^ retinas (Fig. 1c,d,f–i). In contrast inactivation of *amotL1* in adult mice did not have any effect on vessel branching or density (Fig. 1j,k).

The reduced expansion of the vessel network in *amotL1*^ec−/ec−^ animals suggested a defect in cell migration. Luminal endothelial cells convert into tip cells that direct vessel migration and contribute to the development of a multicellular stalk^14^. Our analysis of tip cells did not reveal any obvious defect in tip cell morphology (Supplementary Figure 2a). Furthermore, the number of filopodial extensions per tip cell was not significantly different (Supplementary Figure 2b). However, the total number of tip cells per length unit was lower in *amotL1*^ec−/ec−^ mice, which perhaps could be ascribed to the lower vascular density (Supplementary Figure 2c).

**Figure 2:**
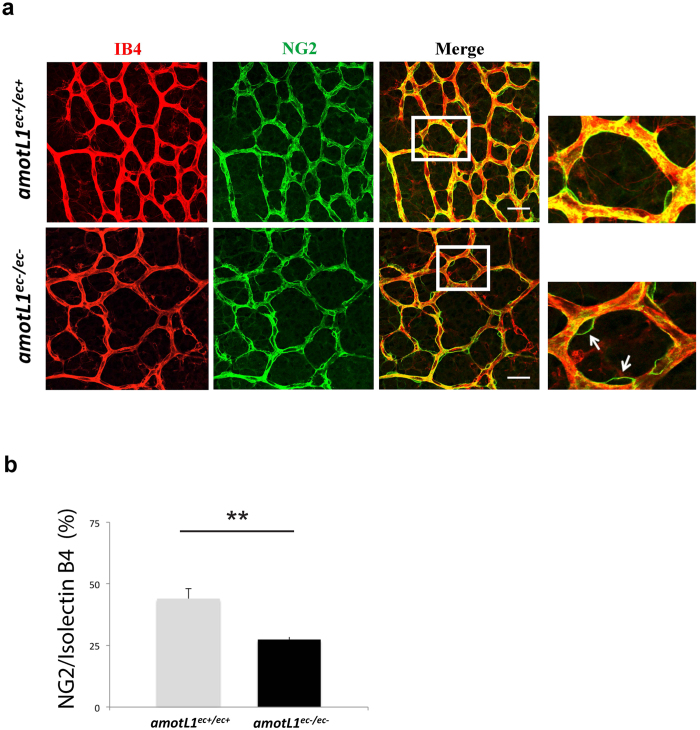
AmotL1 deficiency affects pericyte morphology and coverage of blood vessels. (**a**) Retinas from p6 were stained with IB4 (in red) and anti-NG2 antibodies (in green). Images on the right: magnification of the boxed area. (**b**) The ratio of NG2/IB4 overlap in *amotL1*^ec+/ec+^ and *amotL1*^ec−/ec−^ retinas. ^**^P < 0.005. Size bar, 40 μm.

Endothelial cells depend on other cell types such as microglia, astrocytes and pericytes, to be able to form a functional circulatory network^14^. Each cell type is engaged in distinct functions such as controlling anastomoses (microglia), guiding network branching (astrocytes) and vessel maturation (pericytes). We went on to analyze whether inactivation of *amotL1* affected endothelia-interacting cells in a non-autonomous manner. Whole mount immunostaining of P6 *amotL1*^ec−/ec−^ retinas, performed to visualize microglia and astrocytes did not reveal any striking difference (Supplementary Figure 2d,e). Immunostaining using the NG2 pericyte marker revealed that mutant blood vessels of *amotL1*^ec−/ec−^ retinas were associated with pericytes (Fig. 2a). However, the pericytes showed a decreased area of coverage as the mural cells displayed a changed morphology in that they “bulged” out from the vessels (Fig. 2a,b).

**Figure 3:**
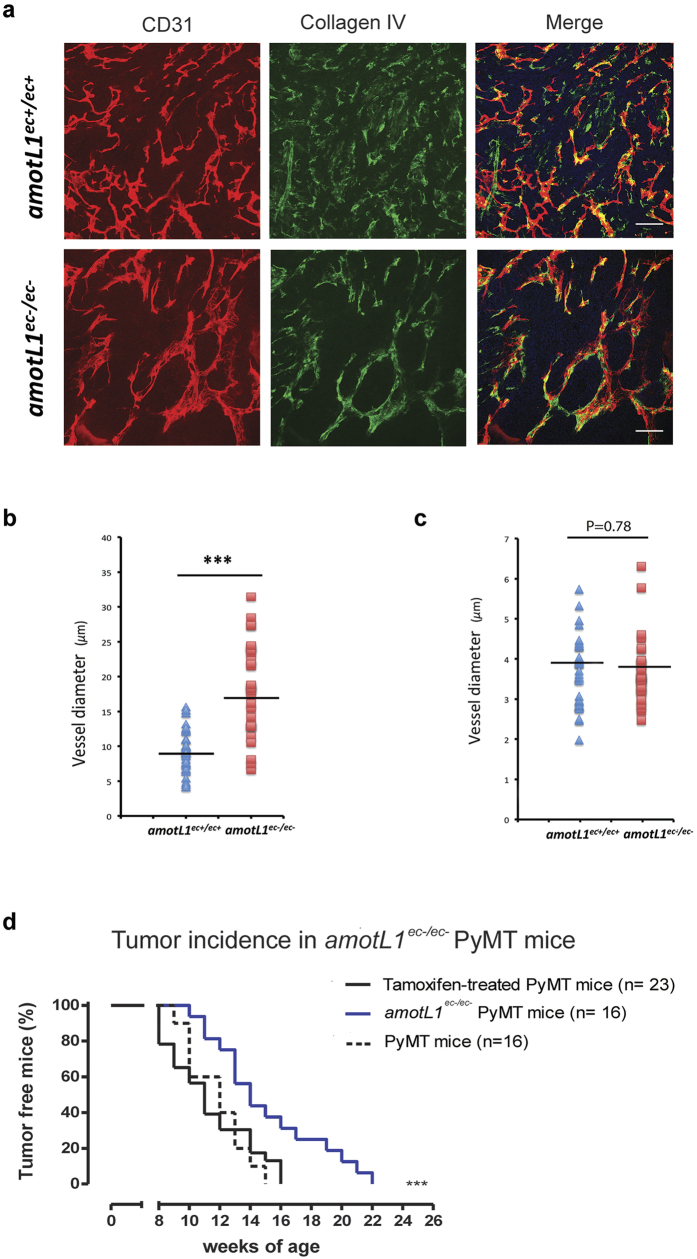
Ablation of *amotL1* in the endothelial lineage in the mouse MMTV-PyMT breast cancer model. (**a**) Tumors derived from *amotL1*^ec+/ec+^ or *amotL1*^ec−/ec−^ MMTV-PyMT mice were stained with antibodies against CD31 (green) or Collagen IV (red) in order to visualize vessel morphology. (**b**) Quantification of vessel diameter of breast tumors from *amotL1*^ec+/ec+^ or *amotL1*^ec−/ec−^ mice. (**c**) Quantification of retinal vessel diameter from adult *amotL1*^ec+/ec+^ and *amotL1*^ec−/ec−^ mice. (**d**) Analysis of breast tumor incidence in MMTV-PyMT mice in *amotL1*^ec+/ec+^ or *amotL1*^ec−/ec−^ background. Size bar, 25 μm.

We then assessed the role of AmotL1 in two tumor model systems, the transgenic model of breast cancer MMTV-PyMT and the Lewis Lung Carcinoma (LLC) transplanted tumor model. In the first model, the MMTV promoter drives the expression of the Polyoma Middle-T oncogene in the mouse mammary epithelium^15^ allowing studies of progressive breast tumor development over a period of 6–14 weeks. The *amotL1*^ec+/ec+^ mice were crossed into the MMTV-PyMT background. Genetic deletion was induced by intraperitoneal tamoxifen injections at week 4. Analysis of the infiltrating blood vessels in the tumor area revealed a marked difference in morphology between the control and AmotL1 deficient mice. *amotL1*^ec−/ec−^ vessels were quite enlarged and dilated as shown by CD31 and Collagen IV immunofluorescent stainings (Fig. 3a and quantification in b). Analysis of normal adult retinal vessels from the same mice showed no vascular defects (Fig. 3c and Supplementary Figure 3). A lower functionality of the tumor vessels was indicated by the longer lag time until tumors were detected in the *amotL1*^ec−/ec−^ mice, whereas tamoxifen administration did not affect tumor progression *per se* (Fig. 3d).

The LLC tumor transplantation model has been extensively used to study tumor vasculature and the efficacy of angiogenesis inhibitors^16^. The LLC tumors grow rapidly and form highly vascularized lesions within 10 days after injection. It has been previously shown that most of the LLC tumor vessels are immature and may regress upon treatment with inhibitors of the VEGF signaling pathway^17^. Here we challenged LLC cells in control and *amotL1*^*f*lox/flox^ mice, as shown in Supplementary Figure 4a, and we assessed that most of the vessels are associated with pericytes (Supplementary Figure 4b). Similar to the transgenic MMTV-PyMT model, ablation of *amotL1* in LLC tumor endothelium resulted in a significant increase in vessel diameter (Supplementary Figure 4c). However, the inhibition of LLC-derived tumors was not significant in amotL1^ec−/ec−^ compared to controls (Supplementary Figure 4d).

**Figure 4:**
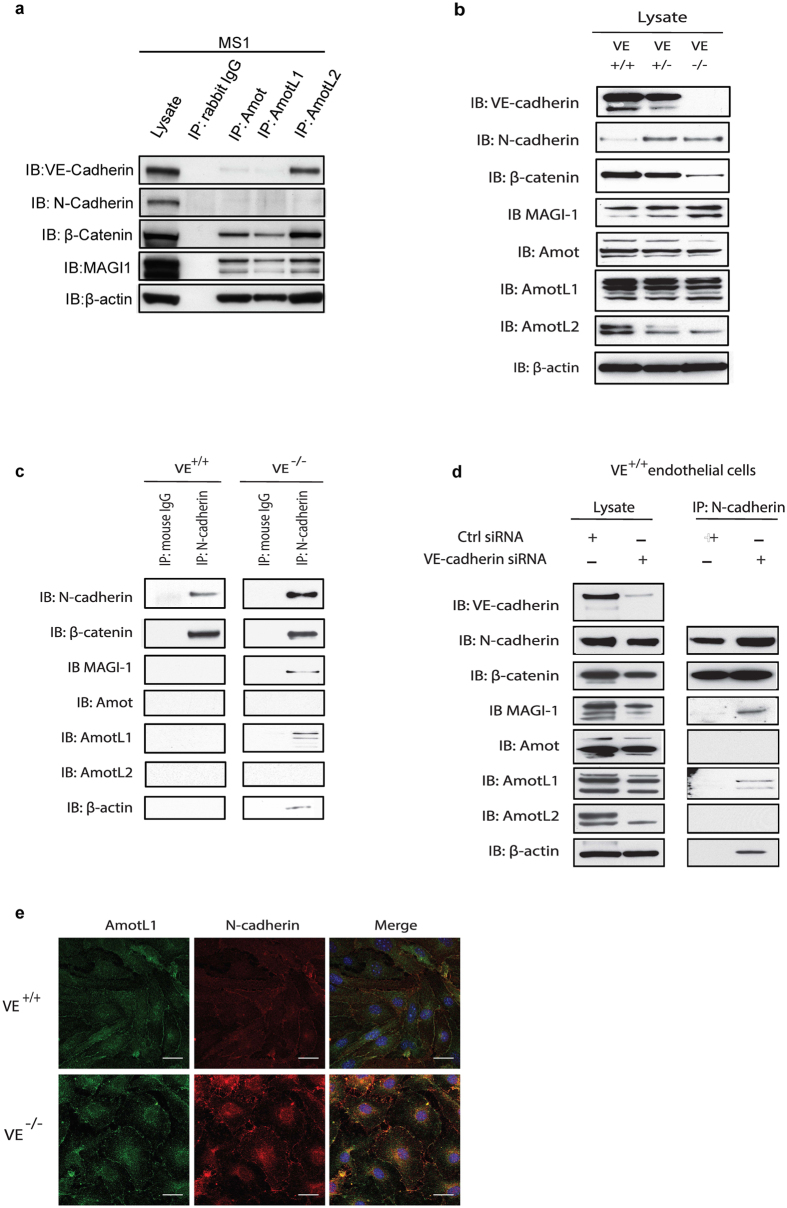
AmotL1 is recruited to N-cadherin associated adhesion complexes in the absence of VE-cadherin. (**a**) Co-immunoprecipitation was performed using antibodies against Amot, AmotL1 and AmotL2 in MS-1 cells. AmotL2 was shown to be associated with VE-cadherin whereas AmotL1 could not be immunoprecipitated with VE-cadherin or N-cadherin. (**b**) Western blot analysis of junctional protein expression in VE-cadherin^+/+^, ^+/−^ and ^−/−^ endothelial cells (**c**) Co-immunoprecipitation of N-cadherin with AmotL1 in VE-cadherin^−/−^ endothelial cells. (**d**) Immunoprecipitation analysis of endothelial cells siRNA depleted of VE-cadherin (**e**) Immunofluorescent staining of AmotL1 (in green) and N-cadherin (in red) in VE^+/+^ and VE^−/−^ cells showing co-localization in cellular adhesion junctions in VE-cadherin deficient endothelial cells. Nuclei were visualized by DAPI staining (in blue). Size bar, 10 μm.

These observations raised the question how ablation of AmotL1 in the endothelial compartment caused vessel enlargement as well as morphological changes in the adjacent pericytes. Endothelial cells and pericytes are closely connected in mature blood vessels where they jointly produce the blood vessel basal membrane. The two distinct cell types communicate by paracrine signaling factors as well as direct physical contact in cell-cell adhesions mediated by N-cadherin. In our previous studies we have shown that AmotL2 is part of the VE-cadherin junctional complex and affects endothelial radial actin filaments and cell morphology in the aorta^9^. Co-immunoprecipitation analysis in mouse endothelial cells showed that VE-cadherin was primarily associated with AmotL2 and weakly with Amot or AmotL1 (Fig. 4a). Endothelial cells also express N-cadherin that is, in contrast to VE-cadherin, not endothelial specific as it is expressed in a number of mesenchymal cells such as pericytes^18^. N-cadherin is thought to participate in vessel stabilization by interacting with peri-endothelial cells during vessel formation and therefore a possible binding partner for AmotL1. However, no association could be detected between AmotL1 and N-cadherin in the co-immunoprecipitation analysis (Fig. 4a). Previous findings have shown that *in vitro* and *in vivo* N-cadherin junctional localization is controlled by VE-cadherin expression level^19^,^20^,^21^. In wild type (wt) endothelial cells *in vitro*, N-cadherin displays a diffuse localization on the cellular membrane whereas in VE-cadherin depleted cells N-cadherin is localized at adherent junctions^22^. It was possible that a homotypic interaction between N-cadherin in different cell types was required for the recruitment of AmotL1 to N-cadherin associated adhesion complexes. The co-immunoprecipitation analysis was repeated in VE-cadherin^−/−^ and VE-cadherin^+/+^ endothelial cells with the result that AmotL1 was being pulled down in complex with N-cadherin only in the VE-cadherin^−/−^ cell line (Fig. 4b,c, Supplementary Figure 5). Similar results were obtained in VE-cadherin siRNA depleted endothelial cells (Fig. 4d). Co-localization of AmotL1 and N-cadherin in cellular junctions was also detected in VE-cadherin^−/−^ cells (Fig. 4e).

**Figure 5:**
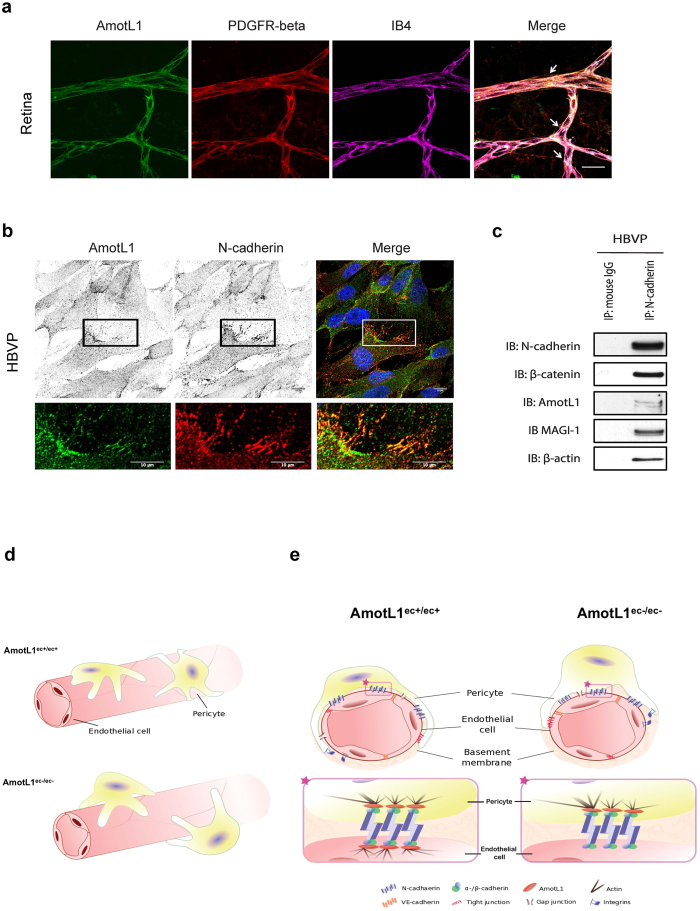
AmotL1 is expressed in pericyte and interacts with N-cadherin. (**a**) IF staining of AmotL1 (in green) in wild type mouse retina at P6. Endothelium was stained by IB4 (in purple) and pericytes were staining by PDGFR-beta (in red). AmotL1 is present in pericyte/endothelial junctions shown by co-localization of AmotL1 and PDGFR-beta (green and purple staining, white arrow). Scale bar, 25 μm. (**b**) Human vascular brain pericytes (HBVP) were stained by AmotL1 and N-cadherin and DAPI (blue) as indicated in the figure. *Bottom:* magnification of the boxed area on the *top.* (**c**) Co-immunoprecipitation of N-cadherin with AmotL1 in HBVP. (**d**) Endothelial AmotL1 deficiency results in decreased pericyte coverage of blood vessels. (**e**) Hypothetical model of how the N-cadherin/AmotL1 protein complex relays mechanical force between endothelial cells and pericytes.

We also analyzed AmotL1 and N-cadherin expression in pericytes. In the developing retina, AmotL1 could be detected in PDGFR-beta positive pericytes associated to IB4-postive retinal blood vessels (Fig. 5a). Immunofluorescent analysis showed overlap between AmotL1 and N-cadherin (Fig. 5b). Furthermore, AmotL1 could be pulled down associated to N-cadherin in human pericytes (Fig. 5c). In conclusion, we show that AmotL1 is a specific partner of N-cadherin in both endothelial cells and pericytes and our data provide evidence that this interaction is important for vascular network organization and proper endothelial/pericytes interaction (Fig. 5d,e).

## Discussion

In this paper we provide insight regarding the role of the scaffold protein AmotL1 in normal and pathological angiogenesis. We show for the first time that AmotL1 is associated to the cellular adhesion molecule N-cadherin. This highlights the importance of Amot proteins as specific interactors of cellular adhesion proteins with distinct roles in controlling the cellular architecture of blood vessels during angiogenesis.

We provide evidence that AmotL1 is essential for the development of a proper vascular network during normal vascularization of the mouse retina. Our previous findings have shown that AmotL2 links the VE-cadherin and confers mechanical force via contractile actin filaments via cell-cell junctions. In contrast, AmotL1 does not associate to VE-cadherin and it is apparently not involved in the homotypic interactions of endothelial cells. In support of this notion, we could show that AmotL1 does not bind VE-cadherin and that knock-down of AmotL1 does not visibly affect actin filaments associated with VE-cadherin. *In vitro*, VE-cadherin mediates adhesion between adjacent endothelial cells and is located at adherens junctions whereas N-cadherin has a diffusely dispersed distribution all over the outer membrane. It has previously been shown that VE-cadherin displaces N-cadherin from endothelial cell junctions and that reduction of VE-cadherin allows N-cadherin to form homotypic cell-cell interactions^19^. In this report, we could only find evidence of AmotL1 binding to N-cadherin in the absence of VE-cadherin. This suggests that the recruitment of AmotL1 is dependent on homotypic N-cadherin interactions in cell junctions. The VE-cadherin-dependent displacement of N-cadherin from endothelial cell contacts allows binding to perivascular N-cadherin expressing cells *in vivo*. Indeed, multiple reports have shown that N-cadherin is required for endothelial-pericyte cross-talk. N-cadherin is typically expressed in cells of mesenchymal origin including vascular mural cells such as pericytes and smooth muscle cells. A shared basal membrane separates mural cells and endothelial cells but these cells make direct contact through cavities in the basal membrane. Several pieces of evidence suggest that N-cadherin is essential for this interaction between endothelial cells and pericytes. Ji-Hye Paik and coworkers have shown that blocking N-cadherin binding in *in vitro* co-cultures abrogated the interaction of endothelial cells and pericytes^23^. Similar results *in vivo* in chick brain suggested that N-cadherin is essential for the interaction of endothelial cells and pericytes and the resulting stabilization of blood vessels^24^. Furthermore, genetic inactivation of N-cadherin resulted in impaired pericyte coverage of endothelial sprouts *in vitro*^25^.

In our experiments, we observed a non-autonomous phenotype in the pericytes of the developing mouse retina. As N-cadherin and AmotL1 are expressed in both endothelial cells and pericytes, it is compelling to speculate that ablation of AmotL1 in the endothelial compartment will affect the pericytes in a non-autonomous way. Indeed, in *amotL1*^ec−/ec−^ retinas, the pericyte coverage was decreased as the pericytes changed shape and bulged out from the vessels.

Extensive data show that endothelial-derived PDGF-B is essential for the recruitment of pericytes in newly formed vessels^26^. Impaired access to PDGF-B through genetic manipulation alters the retinal vascular density and increases tumor blood vessel diameter^27^. This is quite similar to what we have observed in AmotL1 deficiency in endothelial cells during normal and tumor blood vessels development. AmotL1, however, appeared not to be essential for pericyte recruitment as there were no apparent change in the number of pericytes/vessels in the amotl1 ec/ec mice.

TGF-β has long been implicated in the maturation of blood vessels by stimulation of basal membrane components^28^. Recently, Li and coworkers showed that inactivation of the TGF-beta effector protein smad4 in the central nervous system affected the pericytes-endothelial coupling of brain vessels. Interestingly, Smad4 together with Notch signaling affected the expression of N-cadherin. Cerebral capillaries with decreased levels of N-cadherin could maintain the ability to recruit pericytes but failed to form proper adhesions resulting in pericytes detachments^29^. We propose a model by which AmotL1 is recruited to N-cadherin localized to peg-sockets in order to mechanically couple endothelial cells to pericytes via formation of actin filaments the functionally integrating cell-cell junctions with actomyosin contractility. This would be similar to the mechanisms of action of AmotL2, which in complex with VE-cadherin exerts force between endothelial cells via para-cellular actin filaments. We hypothesize that both AmotL1 and AmotL2 functionally integrate mechanical signals via distinct adhesion molecules to modulate vascular morphogenesis.

These findings raise the question whether AmotL1 is a crucial mediator of other cellular processes. Epithelial-mesenchymal transition involves the downregulation of E-cadherin and upregulation of N-cadherin. Expression of N-cadherin on the tumor cells surface facilitates the adhesion to endothelial cells and subsequently extravasation and metastasis^30^. Indeed, a recent study has shown that AmotL1 is upregulated and promotes invasion in breast cancer^31^. Further studies may shed light whether the tumor: endothelial interactions are dependent on mechanical forces relayed by AmotL1.

## Materials and Methods

### Animals

All animal studies were approved by the North Stockholm Animal Ethical Committee. All experiments were carried out in accordance with the guidelines of the Swedish Board of Agriculture. *amotL1*^floxed/foxed^ mice, which carry a loxP-flanked *amotL1* gene, were crossed to Cdh5(PAC)^CreERT2^ and ROSA26-EYFP double transgenic mice in order to perform loss of function studies. The final mouse model was then crossed with mice overexpressing the Polyoma virus Middle T under control of the MMTV promoter (MMTV-PyMT) bred in the animal facility of the Molecular Biotechnology Center (Turin). All mice in this report were in C57BL/6 background.

### Cell culture

Lewis lung carcinoma (LLC) cells were cultured in DMEM medium (GIBCO, Grand Island, NY) supplemented with 10% FCS (GIBCO), 1% glutamine and 1% penicillin/streptomycin (P/S, Sigma-Aldrich). Ms-1 cells were cultured in RPMI-1640 medium (GIBCO) supplemented with 10% FCS, 1% glutamine and 1% P/S. VE^+/+^ and VE^−/−^ cells were cultured culture medium MCDB131 (GIBCO) supplemented with 20% FCS, glutamine (2 mM, Sigma-Aldrich), 1% P/S, sodium pyruvate (1mM, Sigma-Aldrich), heparin (100 μg/ml, from porcine intestinal mucosa; Sigma-Aldrich), and endothelial cell growth supplement (ECGS) (5 μg/ml, made from calf brain, Sigma-Aldrich). The medium for human vascular brain pericytes (HBVP) was purchased from PromoCell (C-28040).

### Western blot

Cells were lysed in the following buffer (50 mM Tris-HCL pH 7.6, 150 mM NaCl, 1 mM EDTA, 1% Nonident P40), 1 x protease inhibitor (Roche, 04693159001). Lysates were prepared with SDS sample buffer (Novex, 1225644) containing 10% sample reducing agent (Novex, 1176192). Proteins were fractionated in a polyacrylamide Bis-Tris 4–12% gradient precast gel (Novex, NP0322BOX). Afterwards, proteins were transferred to a nitrocellulose membrane (Whatman, 10401396). The membrane was blocked in 5% non-fat milk PBS and 0.1% Tween 20 and incubated with the primary antibody overnight at 4 °C. The membrane was thereafter incubated for 1 hour at R.T. with an adequate horseradish peroxidase-conjugated secondary antibody. Labeled proteins were detected with chemiluminescence (ECL; Amersham, RPN2232).

### Immunoprecipitation analysis

Cell lysates were prepared in the buffer (50 mM Tris-HCL pH 7.6, 150 mM NaCl, 1 mM EDTA, 1% Nonident P40, 1 x protease inhibitor (Roche, 04693159001)). Lysates were incubated with protein G sepharose beads (GE, 17-0618-01) for 1.5 hours at 4 °C as pre-cleaning. Afterwards antibodies against AmotL1 or control rabbit immunoglobulins added in the lysates overnight under rotation at 4 °C. Protein G sepharose beads were added for additional 2 hours. Beads were washed 5 times with lyses buffer and heated for 10 min at 95 °C in 2 x LDS sample buffer (Novex, 1225644) containing 10% sample reducing agent (Novex, 1176192). The samples were fractionated by SDS PAGE and subsequently western blotted. Fractions of whole cell lysates were western blotted for evaluation of IP protein input level.

### Retina angiogenesis assay

Mouse retina angiogenesis assay were performed as previously published^12^,^32^. In brief, tamoxifen (Sigma) was injected intraperitoneally into newborn pups at the dose of 50 μg/pup/day from postnatal day P1 to P3. Eyes were dissected at P6 and fixed in 4% paraformaldehyde for whole mount immunostaining. Branching and vessel density was analyzed using the Angiotools software (https://ccrod.cancer.gov/confluence/display/ROB2/Quick+Guide).

### Immunofluorescence staining

Fixed mouse retinas were dissected out from eyeball under microscope. Retinas was permeabilized and blocked in PBS containing 0.3% Triton X-100 and 2% BSA at 4 °C overnight. Primary antibody in pblec buffer (1.0% Triton X-100 plus 0.1 M MgCl_2_, 0.1 M CaCl_2_, 0.01 M MnCl_2_ in PBS) was added to the retina and incubated at 4 °C overnight and subsequently incubated with the fluorescent-conjugated secondary antibody. Digital images were taken by Leica TCS SP5 confocal microscope. Primary antibodies used are as follows: Isolectin B4 (IB4, 1:50), iBA1 (WAKO, 1:500), GFAP (Invitrogen, 1:100), Collagen IV (Millipore, 1:100), rabbit anti-NG2 (Millipore, 1:50), PECAM1 (BD biosciences, 1:100), goat anti-GFP (Abcam, 1:500).

### Xenograft Tumor Model

Tamoxifen was injected intraperitoneally to wild-type as well as *amotL1*^*flox/flox*^ male mice (≥8 mice per group) at age of 6-week-old (1 mg/mouse/day) for 5 days in a row. Approximately, 0.5 × 10^6^ LLC tumor cells were subcutaneously injected into the right flank of the mouse on the last day of tamoxifen injection. Tumor growth was palpated twice per week and tumor volumes were calculated according to the formula: 0.52 × length × width × width. One week before sacrifice, same dosage of tamoxifen was injected intraperitoneally into the tumor bearing mice for 5 days. When tumors reached the diameter of 10 mm, mice were sacrificed and tumors were resected and analyzed by cryohistology.

### *siRNA* transfection

For siRNA transfections cells were seeded the day before transfection on glass slides (BD Falcon Cultureslides BD Biosciences) coated with 1% BD Matrigel Basement Membrane Matrix (BD Biosciences) in growth medium without antibiotics. Just before transfection, growth medium was exchanged for OPTI-MEM I Reduced Serum Media (Invitrogen). Smartpool siGENOME siRNAs (Dharmacon/Thermo Scientific) against VE-cadherin siRNA from Dharmacon (SMARTpool: ON-TARGETplus Cdh5 siRNA, L-041968-01-0005) as well as Non-Targeting siRNA Pool #2 (D-001206-14) were transfected into the cells with Oligofectamine Transfection Reagent (Invitrogen) according to the manufacturers protocol. siRNAs were used at the final concentrations of 0.32M for both control and *AmotL2* siRNAs. Four hours after transfection serum was added to a final concentration of 20%. Cells were allowed to grow for 72 hours prior to evaluation by immunofluorescence staining or western blot.

### Statistical analysis

Statistical analysis of *in vivo* results based on at least four animals and four images from each sample tissue. Comparisons between different groups were performed using the standard two-tailed Student t-test. A value of P < 0.05 was considered as statistically significant (^*^P < 0.05, ^**^P < 0.01 and ^***^P < 0.001).

## Additional Information

**How to cite this article**: Zheng, Y. *et al*. Angiomotin like-1 is a novel component of the N-cadherin complex affecting endothelial/pericyte interaction in normal and tumor angiogenesis. *Sci. Rep.* **6**, 30622; doi: 10.1038/srep30622 (2016).

## Supplementary Material

Supplementary Information
