# Supplementary material for: Angiomotin like-1 is a novel component of the N-cadherin complex affecting endothelial/pericyte interaction in normal and tumor angiogenesis
Source: Sci Rep. 2016 Jul 28;6:30622. doi: 10.1038/srep30622 (PMC4964570; doi:10.1038/srep30622)
Supplement: Supplementary Information [file srep30622-s1.pdf]

Angiomotin like-1 is a novel component of the N-cadherin complex affecting endothelial/pericyte interaction in normal and tumor angiogenesis.

Yujuan Zheng[1], Yuanyuan Zhang[1], Giuseppina Barutello[2], Kungchun Chiu[1], Maddalena Arigoni[2], Costanza Giampietro[3], Federica Cavallo[2], Lars Holmgren[1*]

[1]Department of Oncology and Pathology, Cancer Centrum Karolinska, Karolinska Institutet, SE-17176 Stockholm, Sweden
[2]Department of Molecular Biotechnology and Health Sciences, Molecular Biotechnology Center, University of Turin, 10126 Turin, Italy
[3]Department of Biosciences, Milan University, Via Celoria 26, Milan 20133, Italy and IFOM, the FIRC Institute of Molecular Oncology, Via Adamello 16, Milan 20139, Italy

[*]To whom correspondence should be addressed.

Lars Holmgren,
Department of Oncology and Pathology, R8: 03 CCK, Karolinska Hospital
Lars.Holmgren@ki.se
Cell: +46734036794

Supplementary information

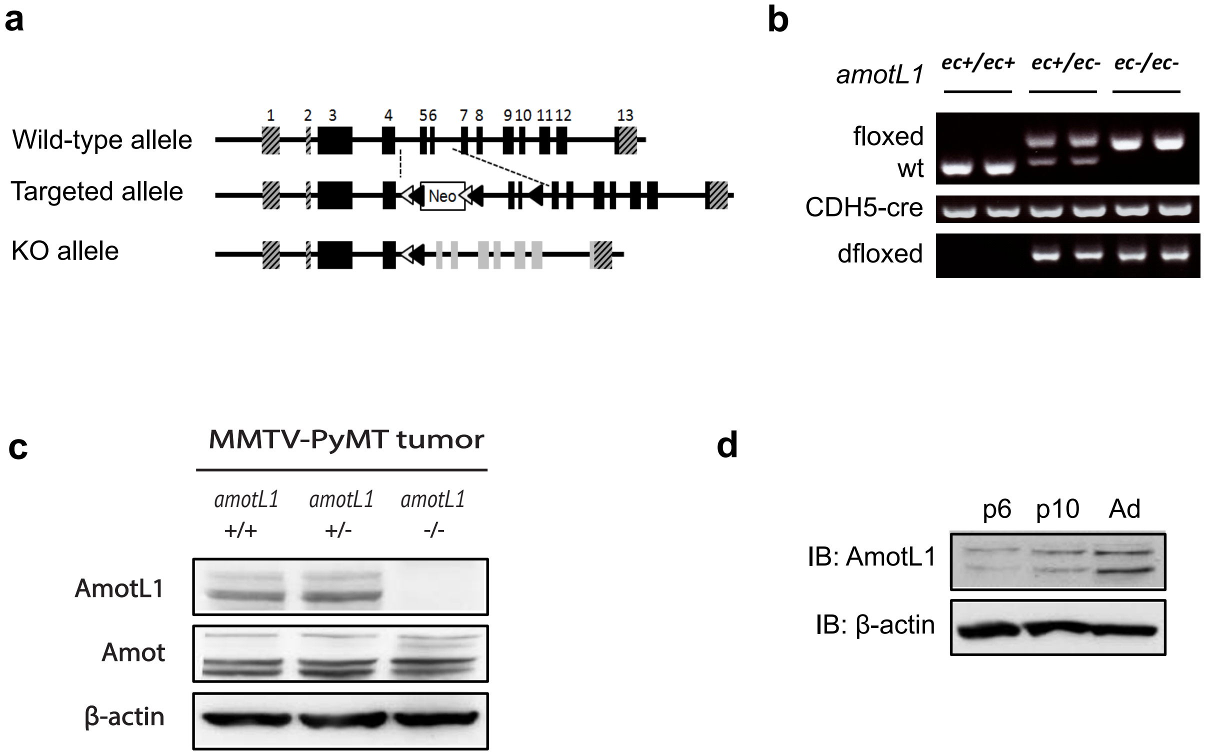

Supplementary Figure 1. Generation of *amotL1*^{floxed}/^{floxed}CDH5(PAC)^{ERT} mice.
(a) Targeting strategy: the exons 5 and 6 of the *amotL1* gene was flanked with LoxP sites which cause a frame shift resulting in premature stop codon at 300 a.a. **(b)** PCR genotyping of *amotL1*^{floxed}/^{floxed} CDH5(PAC)^{ERT} mice abbreviated in the paper as *amotL1*$^{ec-/ec-}$ mice. **(c)** Western blot analysis showing the loss of AmotL1 in *amotL1*$^{floxed/floxed}$/MMTV-Cre/PyMT tumor cells. **(d)** Western blot analysis of *amotL1* expression during the indicated postnatal stages of mouse retinas._

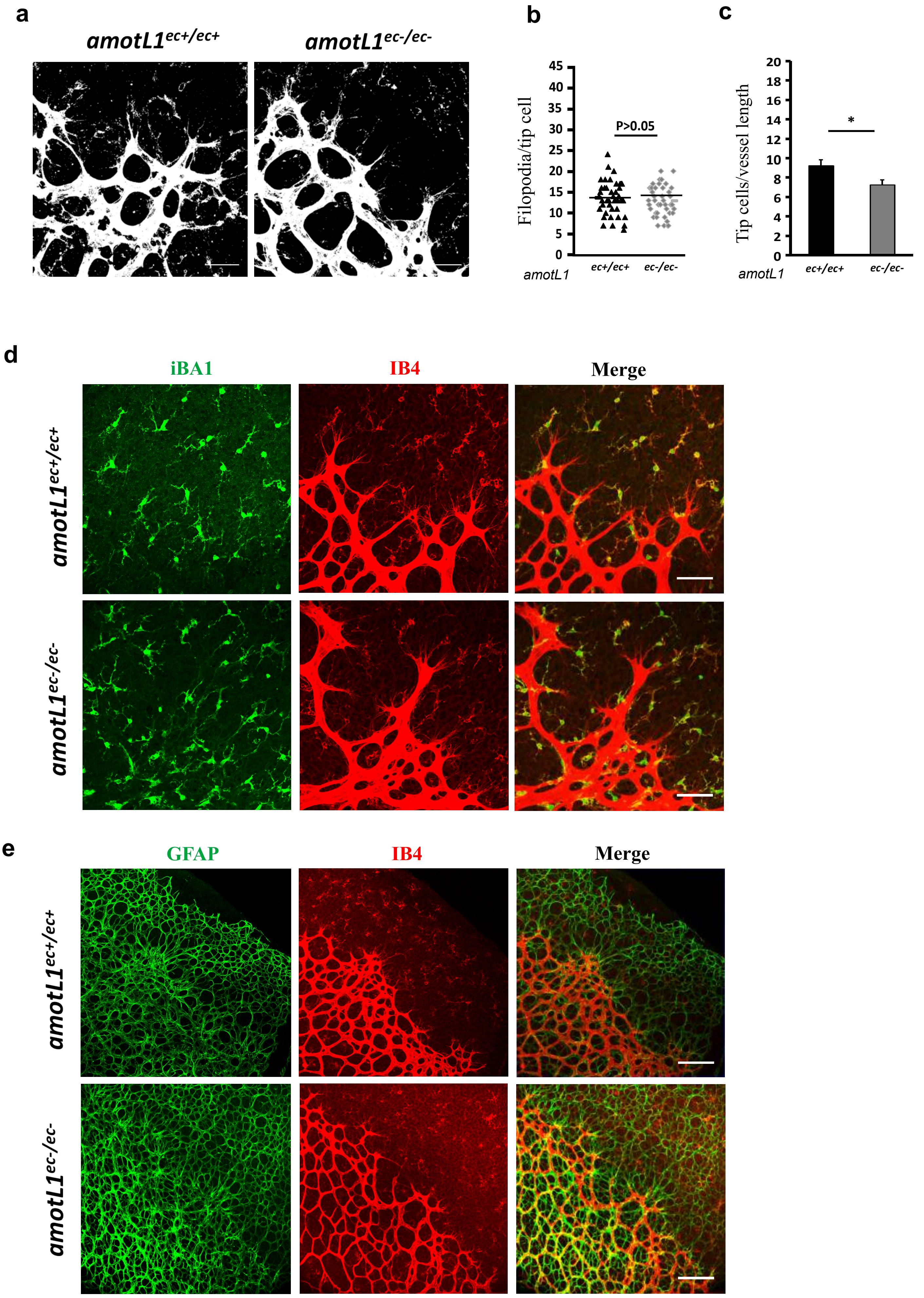
a
amotL1ec+/ec+
amotL1ec-/ec-
b
Filopodia/tip cell
45
40
35
30
25
20
15
10
5
0
P>0.05
amotL1
ec+/ec+
ec-/ec-
c
Tip cells/vessel length
20
18
16
14
12
10
8
6
4
2
0
*
amotL1
ec+/ec+
ec-/ec-
d
iBA1
IB4
Merge
amotL1ec+/ec+
amotL1ec-/ec-
e
GFAP
IB4
Merge
amotL1ec+/ec+
amotL1ec-/ec-

Supplementary Figure 2. Analysis of tip cell, microglia and astrocyte phenotypes after *amotL1* depletion.
(**a**) Panels show P6 retinas stained with IB4 to visualize tip cells and the filopodia at high magnification. (**b**) Analysis of the number of filopodia/cell in *amotL1*$^{ec+/ec+}$ and *amotL1*$^{ec-/ec-}$ tip cells. (**c**) The number of tip cells per length units revealed a small but significant difference. **(d)** Top panel, *amotL1*$^{ec+/ec+}$, and bottom panels *amotL1*$^{ec-/ec-}$, show P6 retinas, microglia cells were visualized by antibodies against iBA1 (green) and blood vessels by IB4 lectin staining (red). **(e)** P6 retinas, astrocytes were visualized using antibodies against GFAP (green) and blood vessels by IB4 (red) staining. *P<0.01. Size bars are (A and D) 25μm and (E) 50μm.

Adult mouse retina

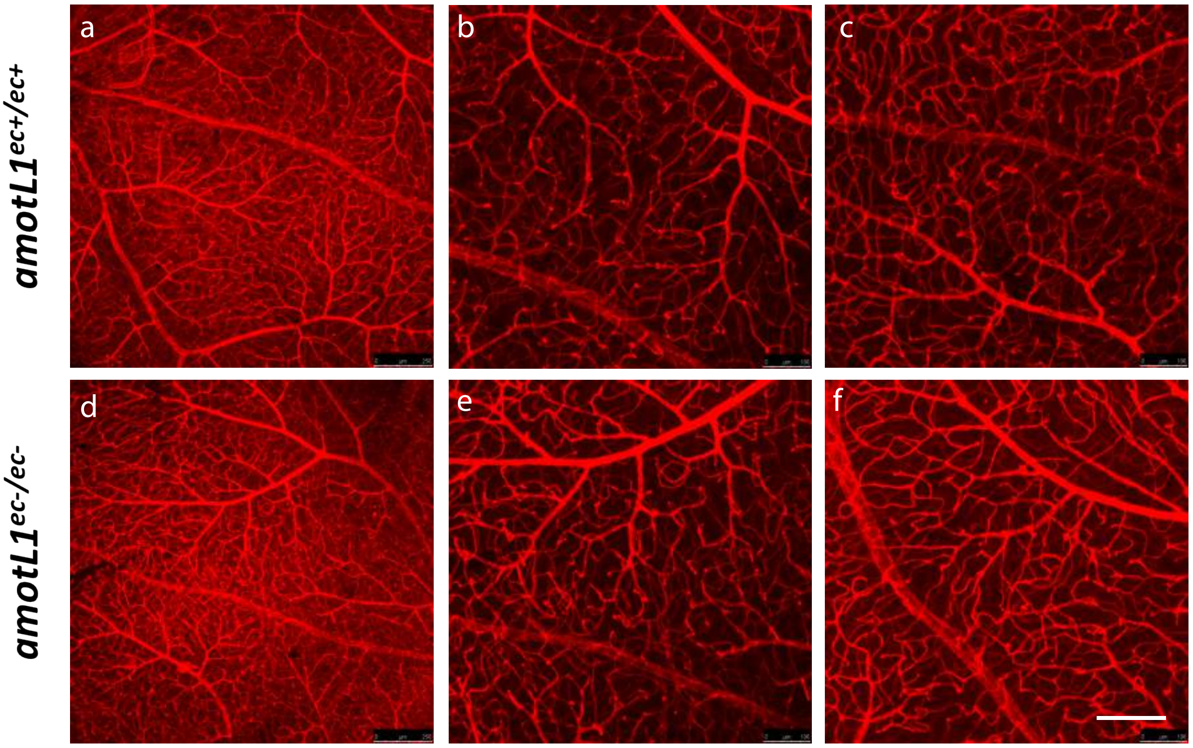

Supplementary Figure 3. Ablation of *amotL1* in adult mouse.
IF staining of IB4 (in red) showing blood vessel network in mouse retina in *amotL1*$^{ec+/ec+}$ and *amotL1*$^{ec-/ec-}$ mice. (**a-c**) Images are from three independent *amotL1*$^{ec+/ec+}$ mice, while (d-f) are from three *amotL1*$^{ec-/ec-}$ mice. Size bar, 25μm.

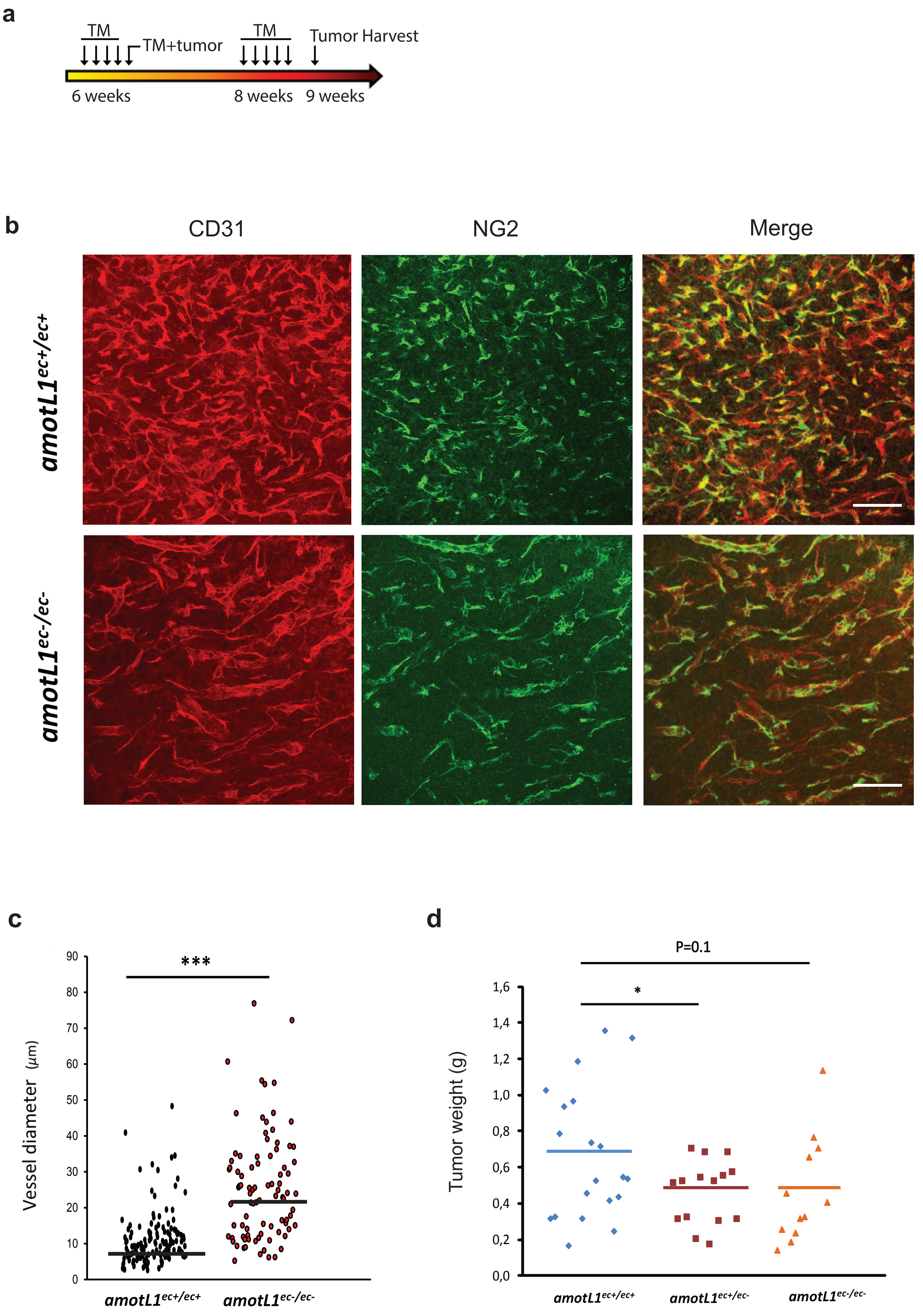
a
TM
TM+tumor
TM
Tumor Harvest
6 weeks
8 weeks
9 weeks
b
CD31
NG2
Merge
amotL1ec+/ec+
amotL1ec-/ec-
c
***
Vessel diameter (µm)
amotL1ec+/ec+
amotL1ec-/ec-
d
P=0.1
*
Tumor weight (g)
amotL1ec+/ec+
amotL1ec+/ec-
amotL1ec-/ec-

Supplementary Figure 4. Effects of endothelial *amotl1* deletion on LLC tumor angiogenesis. **(a)** Strategy used for tamoxifen and LLC tumor injections. (**b**) IF staining of 100 μm vibratome sections of LLC tumors with antibodies against CD31 (in red) and NG2 (in green). (**c**) Quantification of diameter of blood vessels in *amotL1*$^{ec+/ec+}$ and *amotL1*$^{ec-/ec-}$ mice. (**d**) Analysis of tumor weight of LLC tumors grown in *amotL1*$^{ec+/ec+}$, *amotL1*$^{ec+/ec-}$ and *amotL1*$^{ec-/ec-}$ mice. ***P<0.001. Size bar, 25μm.

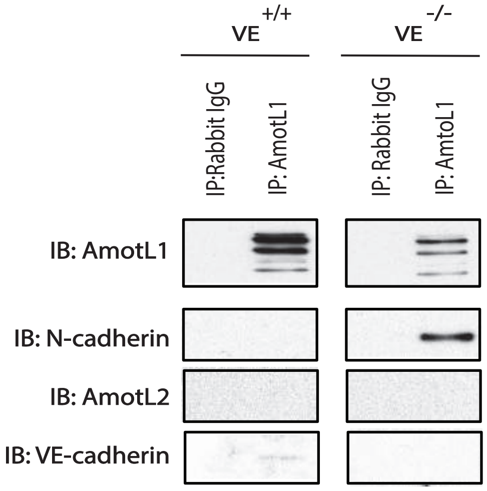

Supplementary Figure 5. AmotL1 is associated to N-cadherin in the absence of VE-cadherin. Co-immunoprecipitation was performed using antibodies against AmotL1 in VE-cadherin $^{+/+}$ and $^{-/-}$ endothelial cells.

Figure 4b

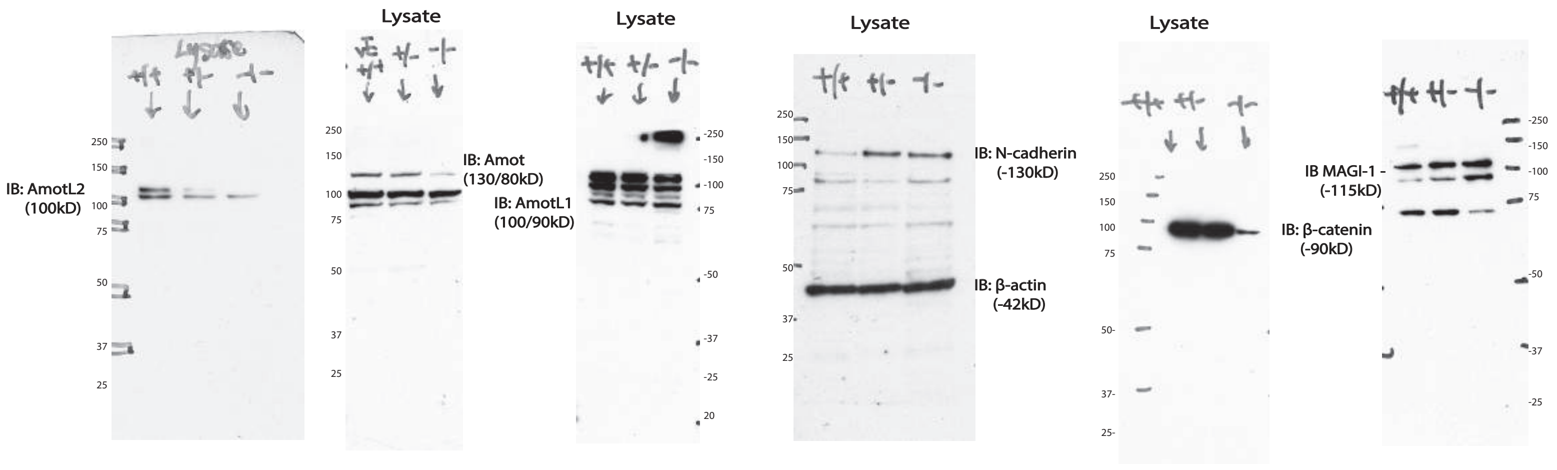

Figure 4c

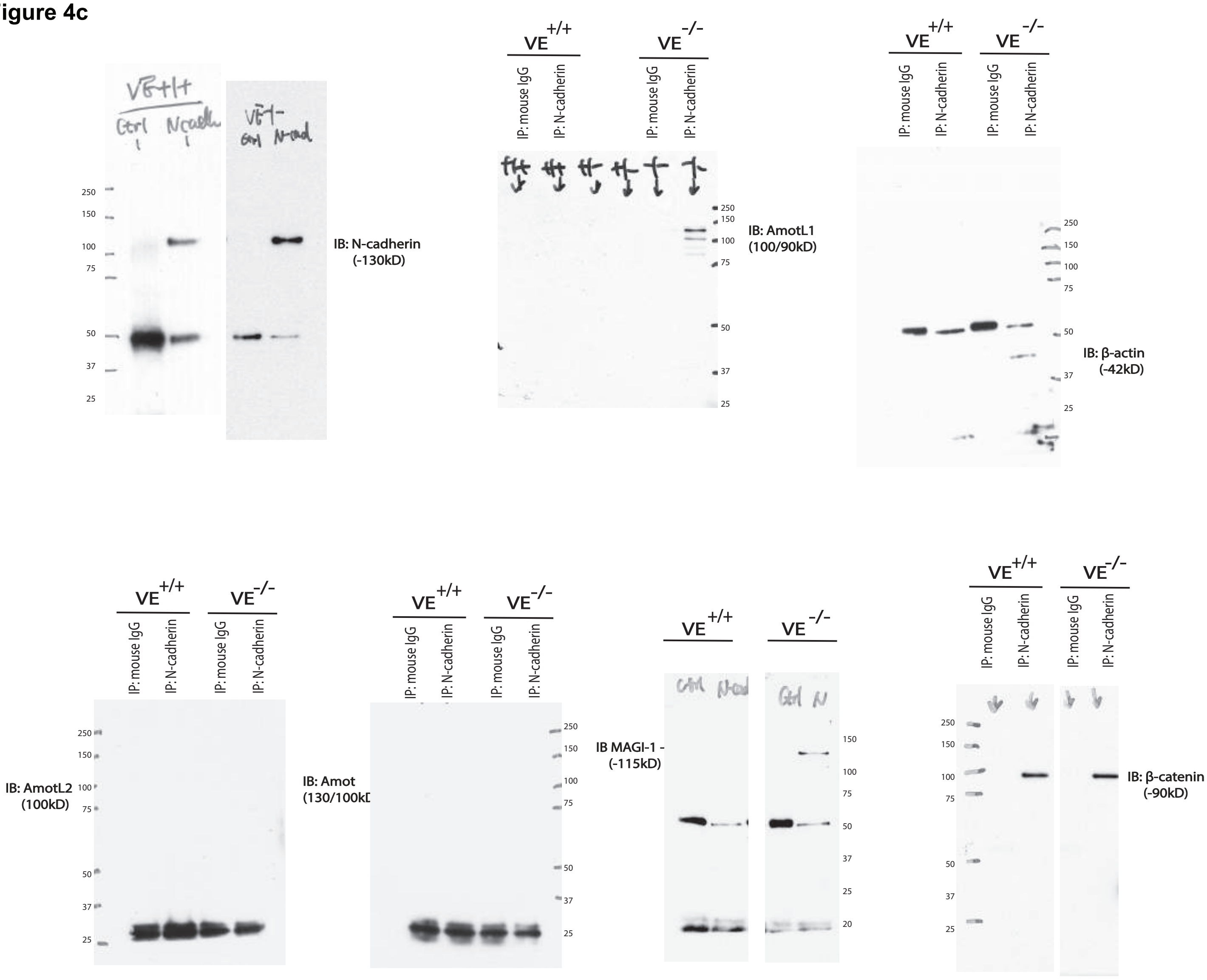

Supplementary Figure 6. Full length blots to Figure 4b and 4c

Figure 4d

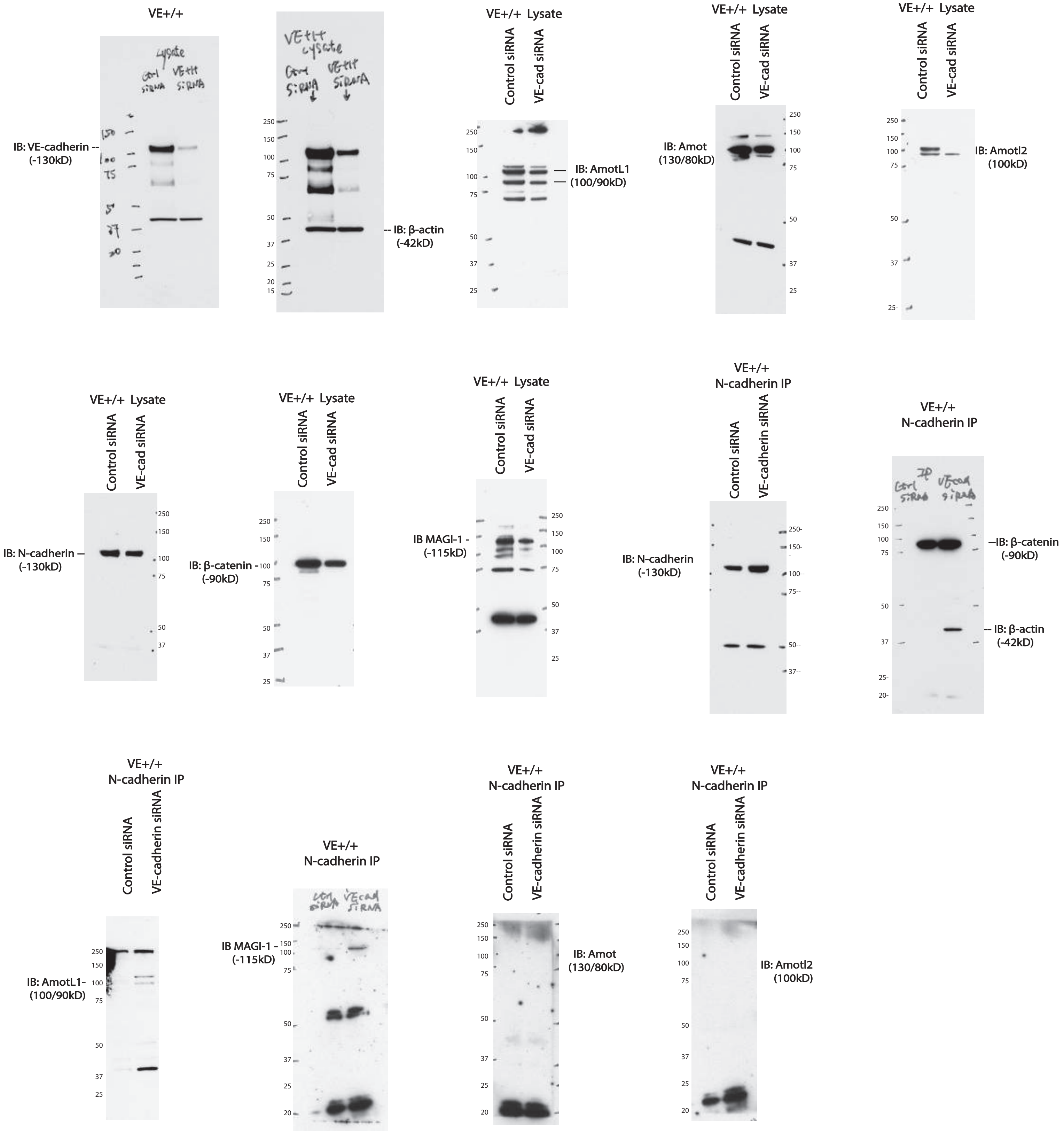

Supplementary Figure 7. Full length blots to Figure 4d

Figure 5c

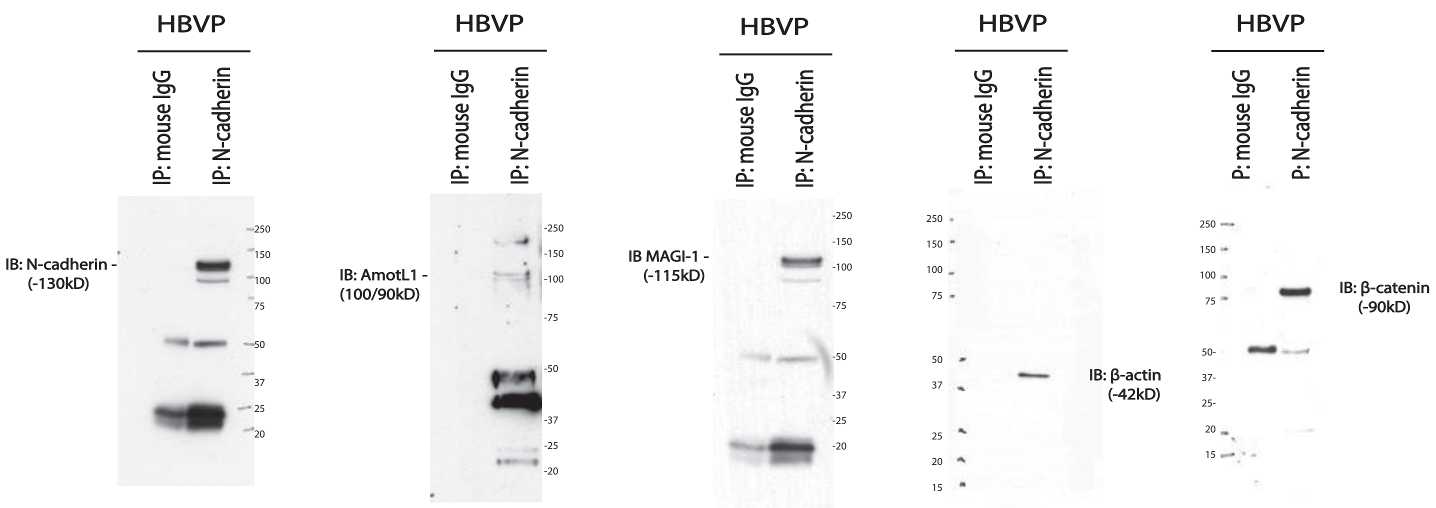

Supplementary Figure 1c

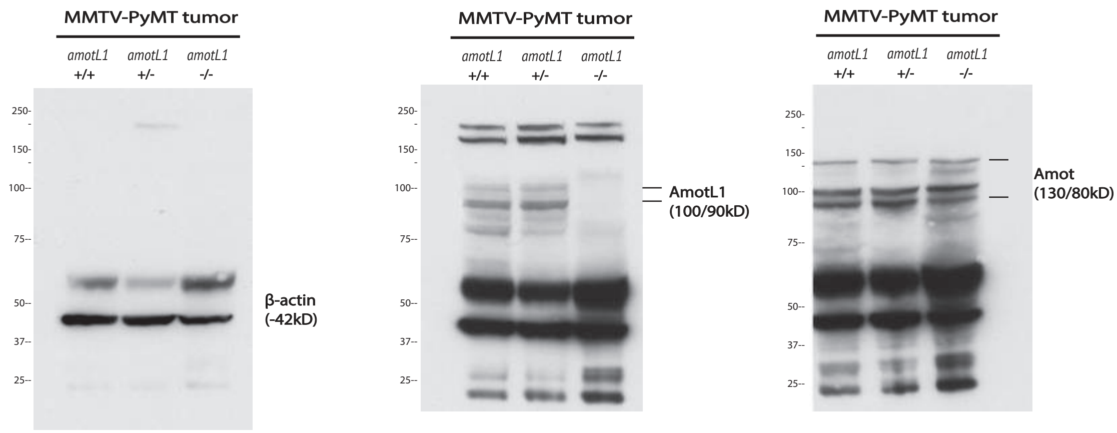

Supplementary Figure 1d

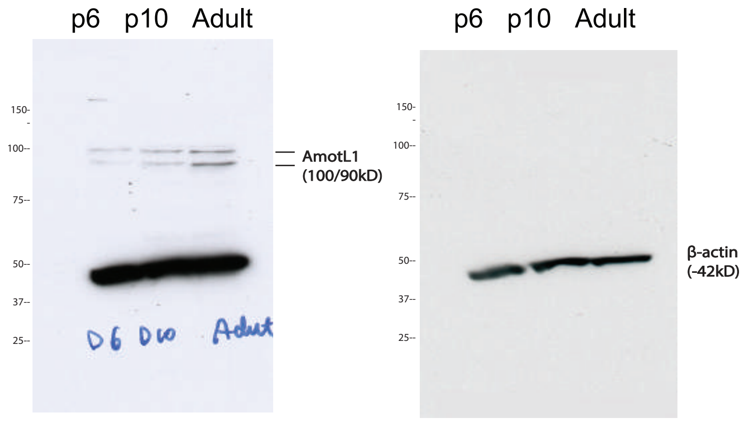

Supplementary Figure 8. Full length blots to Figure 5c, Supplementary Figure 1c and 1d.

Supplementary Figure 5

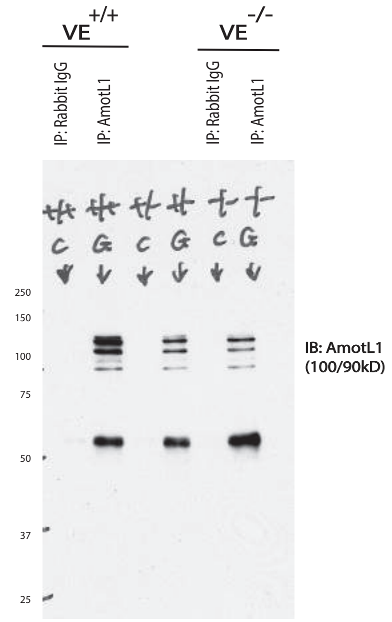

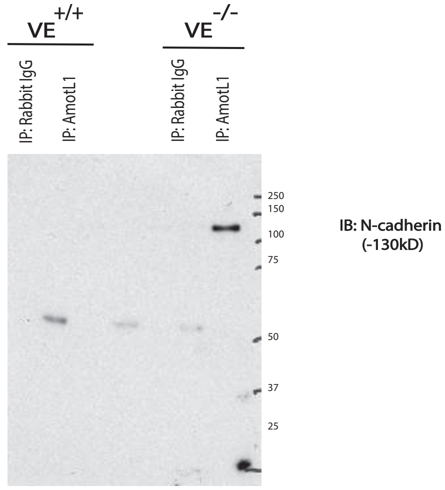

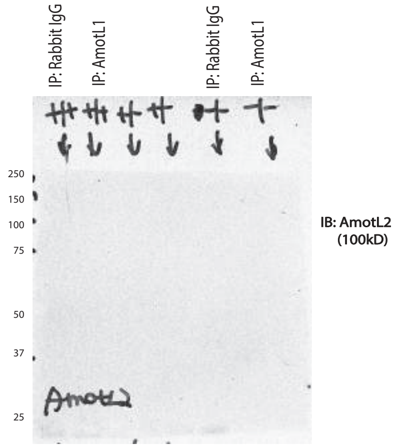

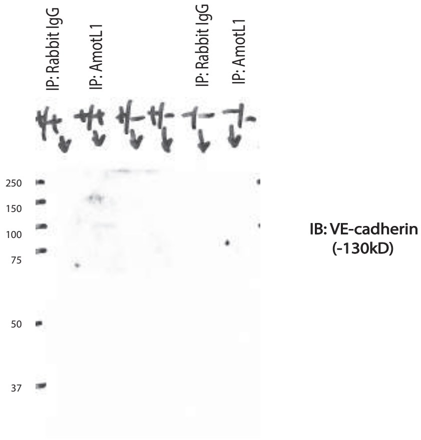

Supplementary Figure 9. Full length blots to Supplementary Figure 5
